# Collective Structural Changes in Vermiculite Clay Suspensions Induced by Cesium Ions

**DOI:** 10.1038/srep06585

**Published:** 2014-10-10

**Authors:** Ryuhei Motokawa, Hitoshi Endo, Shingo Yokoyama, Shotaro Nishitsuji, Tohru Kobayashi, Shinichi Suzuki, Tsuyoshi Yaita

**Affiliations:** 1 Actinide Coordination Chemistry Group, Quantum Beam Science Center (QuBS), Japan Atomic Energy Agency (JAEA), Tokai, Ibaraki 319-1195, Japan; 2 Neutron Science Division, Institute of Material Structure Science, High Energy Accelerator Research Organization, 203-1 Shirakata, Tokai, Ibaraki 319-1106, Japan; 3 Department of Material Structure Science, The Graduate University for Advanced Studies (SOKENDAI), 203-1 Shirakata, Tokai, Ibaraki 319-1106, Japan; 4 Central Research Institute of the Electric Power Industry, Abiko, Chiba 270-1194, Japan; 5 Graduate School of Science and Engineering, Yamagata University, Yonezawa, Yamagata 992-8510, Japan

## Abstract

Following the Fukushima Daiichi nuclear disaster in 2011, Cs radioisotopes have been dispersed over a wide area. Most of the Cs has remained on the surface of the soil because Cs^+^ is strongly adsorbed in the interlayer spaces of soil clays, particularly vermiculite. We have investigated the microscopic structure of an aqueous suspension of vermiculite clay over a wide length scale (1–1000 Å) by small-angle X-ray scattering. We determined the effect of the adsorption behavior of Cs^+^ on the structural changes in the clay. It was found that the abruption of the clay sheets was induced by the localization of Cs^+^ at the interlayer. This work provides important information for predicting the environmental fate of radioactive Cs in polluted areas, and for developing methods to extract Cs from the soil and reduce radioactivity.

Following the Fukushima Daiichi nuclear disaster in 2011, a considerable amount of research has focused on removing radioactive cesium isotopes (^134^Cs and ^137^Cs) from soil and reducing radioactivity in contaminated areas^1^,^2^,^3^,^4^,^5^,^6^,^7^,^8^,^9^,^10^,^11^. Cs^+^ interacts strongly and selectively with phyllosilicate soil fractions, such as vermiculite and smectite^12^,^13^,^14^,^15^,^16^,^17^,^18^, which consist of a layered structure of 2:1 phyllosilicate clay. Cs^+^ rigidly packs into the center of the six-membered rings in the upper and lower SiO_4_ tetrahedral sheets across the interlayers, where the characteristic configuration is attributed to the high affinity between the Cs^+^ and the clay. In particular, Cs^+^ desorption from vermiculite is difficult because Cs^+^ binds to the clay interlayer spaces semipermanently^19^,^20^. The frayed-edge planar site conceptual model is often used to explain the high affinity of Cs^+^ for clay^11^,^20^,^21^,^22^,^23^,^24^,^25^, although there is no direct experimental evidence for the structure. The relationship between Cs^+^ adsorption to the clay and the clay microscopic structure remains unexplained in many respects and has yet to be studied more closely based on empirical data.

Ionic exchange of Cs^+^ with the hydrated or dehydrated alkali metal and alkaline earth metal cations^26^,^27^ that originally occupy the clay interlayer space probably occurs in suspensions. Therefore, investigating the microscopic structure of vermiculite in suspension and the structural changes induced by Cs^+^ adsorption should provide important information for treating contaminated soil. In contrast to electron microscopy techniques, X-ray scattering can be used to observe clay suspensions without pre-treatments that require drying. Moreover, the scale of the small-angle X-ray scattering (SAXS) measurements in this study was between 1 and 1000 Å, which corresponds well to the scale of the layer structure and its spatial arrangement in the clay crystal domain. In particular, the microscopic structure of clay suspensions on submicron-length scales has not yet been quantitatively examined.

In this study, we aimed to investigate the microscopic structure of vermiculite clay suspensions with Cs^+^ as a function of the adsorbed Cs^+^ concentration and elucidate the relationship between the structure of vermiculite and the adsorption of Cs^+^ to vermiculite. These findings provide important fundamental information about the stability and desorption of Cs^+^ on clay particles. Therefore, our study should contribute to (i) predicting the environmental fate of radioactive cesium in contaminated areas, and (ii) developing a method to extract radioactive cesium from contaminated soil. Here, we report and discuss the SAXS profiles obtained for vermiculite suspensions containing adsorbed Cs^+^ in conjunction with the adsorption behavior of Cs^+^ 23,24,28,29.

## Results

### SAXS investigation of cesium adsorbed vermiculite

Vermiculite clay, obtained from Fukushima, Japan, was sonicated to fragment it in suspension. The dried clay powder (200 mg *w*_verm_), was dispersed in aqueous CsCl solutions (20 mL) of five Cs^+^ concentrations ([Cs^+^]_ini_) from 1 to 1000 ppm (see Table 1) for one month. The vermiculite clay initially incorporated hydrated Mg^2+^ in the interlayer spaces. The Mg^2+^ originally occupying the interlayer spaces should exchange with Cs^+^ as [Cs^+^]_ini_ increases. The amount of Cs^+^ adsorbed to the vermiculite clay, evaluated by inductively coupled plasma mass spectrometry (ICP-MS), increased with [Cs^+^]_ini_. Each suspension was directly measured by SAXS. The observed X-ray scattering intensities as a function of the magnitude of the scattering vector *q* [ = (4π/*λ*)sin(*θ*); *λ* is the incident X-ray wavelength and 2*θ* is the scattering angle], *I*_obs_(*q*), were normalized by time and the second moment of the intensity, *Q*, which is proportional to the mean square scattering length density fluctuation^30^,^31^, 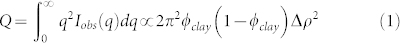
Here, *ϕ*_clay_ is the volume fraction of the clay particles. Δ*ρ* is the scattering contrast between the clay and the solvent and Δ*ρ* = (*ρ*_clay_ - *ρ*_water_) where *ρ*_clay_ and *ρ*_water_ are the scattering length density of clay and of water. In each sample, *ϕ*_clay_ was not known because of sedimentation; therefore, *I*_nor_(*q*) ( = *I*_obs_(*q*)/*Q*) was used to remove the effect of the volume fraction. Thus, we could compare the change in intensity as a function of *w*_Cs_*,* which is the weight of Cs^+^ adsorbed to 200 mg of vermiculite clay (see Table 1).

**Figure 1:**
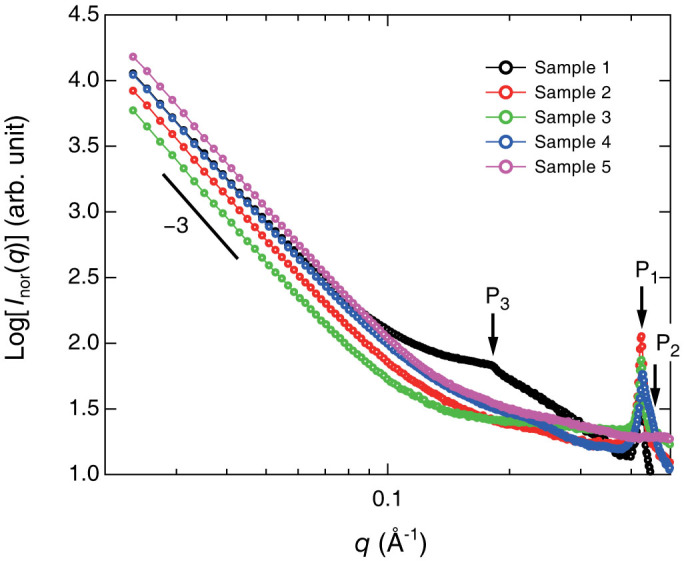
Structural changes in the vermiculite/Cs^+^ suspension on the nano scale. Double-logarithmic plots of the SAXS profiles obtained for Cs^+^ adsorbed on vermiculite in aqueous suspension at different *w*_Cs_ for samples 1 (black circles), 2 (red circles), 3 (green circles), 4 (blue circles), and 5 (pink circles). The peaks indicated by P_1_ and P_2_ originate from the interference between the stacked clay sheets. The peak indicated by P_3_ is attributed to long-range inhomogeneity in the stacking of the clay sheets.

Figure 1 shows *I*_nor_(*q*) as a function of *q* for samples 1–5 (see Table 1) on a double logarithmic scale. The profiles reflect the structural changes in the vermiculite clay induced by the exchange of Mg^2+^ with Cs^+^ in suspension. In the low-*q* region of *q* < 0.1 Å^−1^, the scattering intensity starts to increase in accordance with the power law for scattering, *I*(*q*) ≈ *q*^−*β*^ with *β* ≈ 3. In the high-*q* region of *q* > 0.3 Å^−1^, the scattering maxima at around *q*_m_ = 0.42 Å^−1^, indicated by P_1_ in Fig. 1, are observed. The peaks arise from the interference between the stacked clay sheets^18^,^28^. The distance between neighboring clay sheets (*D* = 2π/*q*_m_) can be estimated as approximately 14.9 Å in samples 1–3. The Bragg diffraction peak shows long tailing toward higher *q* in sample 4. This shoulder peak, (P_2_ in Fig. 1), originates from the interlayer spaces intercalating with the partially dehydrated Cs^+^. In the SAXS profile of sample 5, this peak shifts to a higher value of *q* and its intensity decreases. The peak is hardly visible around *q* = 0.49 Å^−1^. This abrupt decrease in the intensity is attributed to the large distribution of *D* and the decrease of the stacking number, *N*, per crystal domain.

Only sample 1 shows a broad peak at *q* = 0.18 Å^−1^ (arrow and P_3_ in Fig. 1). This peak is attributed to long-range inhomogeneity in the stacking state of the 2:1 phyllosilicate clay sheets, and the length of the inhomogeneity can be roughly calculated as 35 Å from the peak position. The origin of this broad peak is the swollen interlayer spaces in the stacked clay sheets. These spaces can be formed in the crystal domains at a lower Cs^+^ adsorption (*w*_Cs_ = 0.018 mg).

### Scattering model for clay suspension

Because vermiculite clay is a 2:1 phyllosilicate multilayer composed of two tetrahedral sheets and one octahedral sheet, it is reasonable to assume that the scattering intensity at the small-angle region is increased by stacked thin sheets. The small-angle scattering intensity, *I*(*q*), can be described by 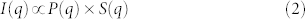
where *P*(*q*) is the form factor, which represents the shape of a single sheet^32^,^33^, and *S*(*q*) is the structure factor for one-dimensionally overlapping plates perpendicular to the plane^32^,^34^,^35^,^36^. It is assumed that the clay sheet is a disk with a base of radius *R* and thickness *d*; therefore, *P*(*q*) is given by 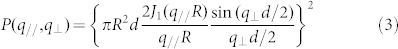
where *J*_1_(*x*) is the cylindrical Bessel function of the first order, and *q*_//_ and *q*_⊥_ are scattering vectors pointing in the in-plane and out-of-plane directions to the base, respectively. The structure factor describes a one-dimensional array of *N* sheets at interval distance *D*, 
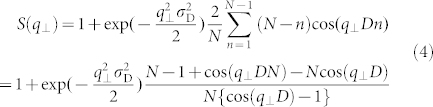
where *σ*_D_ is the standard deviation of each position based on the Gaussian distribution^35^,^36^,^37^. We assume that the stacked layers form particles and the orientation of the particles is random. In this case, the observed intensity is expressed by 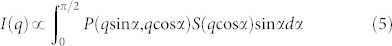
Here, α is the angle between a reference axis and the principal axis of the layers^33^. To evaluate the data quantitatively, the resolution of the instrument and the distributions of *R* and *D* are included.

### Quantitative analyses of SAXS intensity distributions

**Figure 2:**
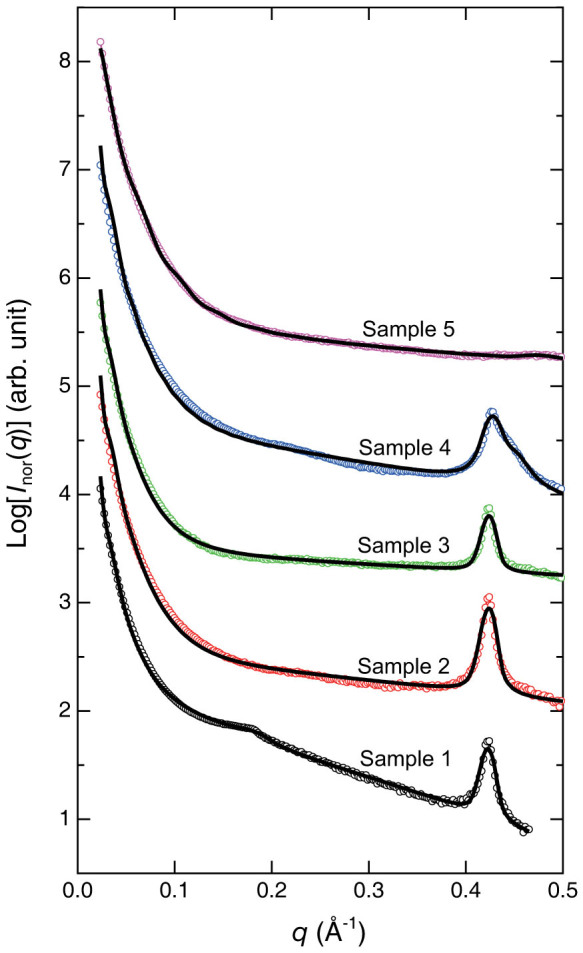
Numerical analyses of SAXS intensity distribution. The SAXS profiles are vertically shifted to avoid overlap by an offset constant of 1, and the constant values for samples 1–5 are 0, 1, 2, 3, and 4, respectively. The scattering profile of sample 1 is shown on a net intensity scale of log[*I*_nor_(*q*)]. The solid black lines are best-fit theoretical profiles obtained by using equation (2) together with the characteristic parameters and error (Table 2).

The solid lines in Fig. 2 show the best-fit theoretical scattering curves. The theoretical profiles reproduce the experimentally observed SAXS profiles well over a wide *q*.

The power law scattering with the exponent *β* ≈ 3 at low *q* (*q* < 0.1 Å^-1^) is related to two parameters, *R* and *N* (equations (3) and (4)). When *R* or *N* increases, the scattering intensity in the low-*q* region tends to shift further toward the low-*q* region, because the size of the scatterer becomes large. *N* also affects the peak width; as *N* increases, the peak width narrows. Therefore, *R* and *N* must be evaluated simultaneously for quantitative data analysis.

For double Bragg peaks (samples 1 and 4), equation (4) can be modified as 
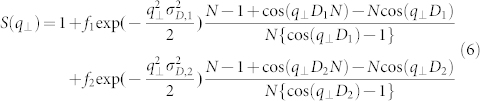
where *D*_1_ and *D*_2_ are the interlayer distances, and *σ*_D,1_ and *σ*_D,2_ are the corresponding standard deviations^37^ in the single crystal domain. We assume that random distribution of the different spaced layers, and the fractions are given by *f*_1_ and *f*_2_ (*f*_1_ + *f*_2_ = 1), respectively. Only one Bragg peak is observed in samples 2, 3, and 5, and *f*_1_ = 1 and *f*_2_ = 0 in equation (6) for these samples. The distribution of *D*_1_ and *D*_2_ is calculated from the Gaussian profile of the standard deviation, *σ*_T,1_ and *σ*_T,2_, respectively.

The parameters *R*, *σ*_R_, *N*, *f_i_*, *D_i_*, *σ*_T,*i*_, and *σ*_D,*i*_ (*i* = 1 or 2) were refined to give the best fit to the experimental SAXS profiles (Table 2). The thickness of the clay sheet, *d*, was fixed as 6.6 Å in this numerical analysis. This is because the two tetrahedral clay sheets and one octahedral clay sheet that form the unit of the 2:1 phyllosilicate vermiculite clay sheet have an exact thickness of 2.2 Å^38^. The polydispersity of *N* was not required in order to fit the data. The solid lines in Fig. 2 show the best-fit theoretical scattering curves.

## Discussion

**Figure 3:**
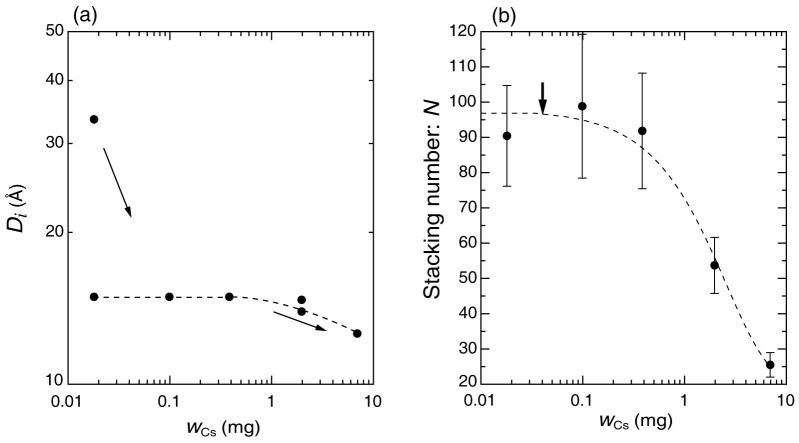
Structural changes in the crystal domain of vermiculite. (a) *D_i_* and (b) *N* with the error plotted as a function of *w*_Cs_ on logarithmic and linear scales, respectively. Dashed lines in (a) and (b) are a visual guide and single exponential function, exp(-*w*_Cs_/*w*_Cs_*), respectively. The arrow in (a) is a visual guide. Errors in *D_i_* in the numerical SAXS analyses are within ±3% accuracy. Error bars of *D_i_* are omitted for clarity.

Figure 3 shows the extracted parameters *D_i_* and *N* plotted as a function of *w*_Cs_ to characterize the structural changes in the crystal domain of vermiculite clay. In Fig. 3a, two characteristic distances, *D*_1_ = 14.9 Å and *D*_2_ = 33.1 Å, are observed at *w*_Cs_ = 0.018 mg (sample 1), where *f*_1_ and *f*_2_ are 0.73 and 0.27, respectively. One component, containing a large swollen interlayer space ( = 33.1 Å; Table 2) in the crystal domain, disappears as the [Cs^+^]_ini_ increases, and only the other component remains in the SAXS profile at *w*_Cs_ = 0.099 mg (sample 2) and *D*_1_ remains as 14.9 Å. This indicates that only large swollen layers shrink, because the values of *N* for samples 1 and 2 are virtually unchanged (Fig. 3b and Table 2). Therefore, we expect that at lower [Cs^+^]_ini_, some Cs^+^ preferentially intercalates in the wide swollen interlayer spaces in addition to adsorption at the edge or planar sites in the crystal domains, whereas the narrower interlayer spaces are not affected.

At *w*_Cs_ = 1.97 mg (sample 4), we estimate that the distance between neighboring sheets decreases in about 20% of the layers (Table 2; *D*_2_ = 14.0 Å, *f*_2_ = 0.21), whereas *D*_1_ is 14.7 Å for *f*_1_ = 0.79. Two peaks are clearly visible, which is evidence of the collective intercalation of Cs^+^. Accordingly, the exchange between the partially dehydrated Cs^+^ and hydrated Mg^2+^ should proceed in the selective interlayers above *w*_Cs_ = 0.384 mg (sample 3). At *w*_Cs_ = 6.94 mg (sample 5), *D*_1_ is 12.6 Å. It has been reported that the value of *D*_1_ decreases to approximately 10.5 Å for the dry powder^28^, which may be partially swollen in suspensions.

The small-angle scattering intensities and the power law below *q* = 0.1 Å^-1^ increases with [Cs^+^]_ini_ from sample 3 to 5 (Fig. 1). Our quantitative analyses suggest that this is caused by the decrease in the stacking number, *N*, at *w*_Cs_ > 0.384 mg (Fig. 3b), whereas *R* is similar in all samples.

Therefore, the changes in both the interlayer space and the stacking number probably originate from the cationic exchange of hydrated Mg^2+^ with partially hydrated Cs^+^. The shrinkage may induce local strain and defects in the crystal domains, which leads to the segmentation of the stacking clay sheets. Next, we describe the effect of the electrostatic interactions in the layer space in relation to Cs^+^ adsorption. The direct coordination of a partially dehydrated Cs^+^ to oxygen atoms in the SiO_4_ tetrahedral sheet, which has a negative charge, locally shields the layer charge of the SiO_4_ tetrahedral sheet^23^,^24^,^29^. This reduces the local electron charge density on the opposite surface of the SiO_4_ tetrahedral sheet. The attractive interaction between the SiO_4_ tetrahedral sheet and hydrated Mg^2+^ is weakened, further swelling occurs in the layer space, and partial abruption takes place in the hydrated Mg^2+^ layer spaces. Consequently, the crystal domain undergoes segmentation and *N* (the grain size of the crystal domain) should decrease (Fig. 3b) when a sufficient amount of Cs^+^ intercalates in the local layer space. The segmented smaller-crystal domain generates new planar regions, which are covered by hydrated Mg^2+^. This planar region is expected to serve as a specific adsorption site for Cs^+^, in addition to the interlayer spaces. Two Cs ions can coordinate directly to an oxygen atom in the SiO_4_ tetrahedral sheets through the exchange with one hydrated Mg^2+^, implying that Cs^+^ intercalation in the swelling layer space of vermiculite clay promotes further Cs^+^ adsorption at the freshly generated crystal domain interface.

**Figure 4:**
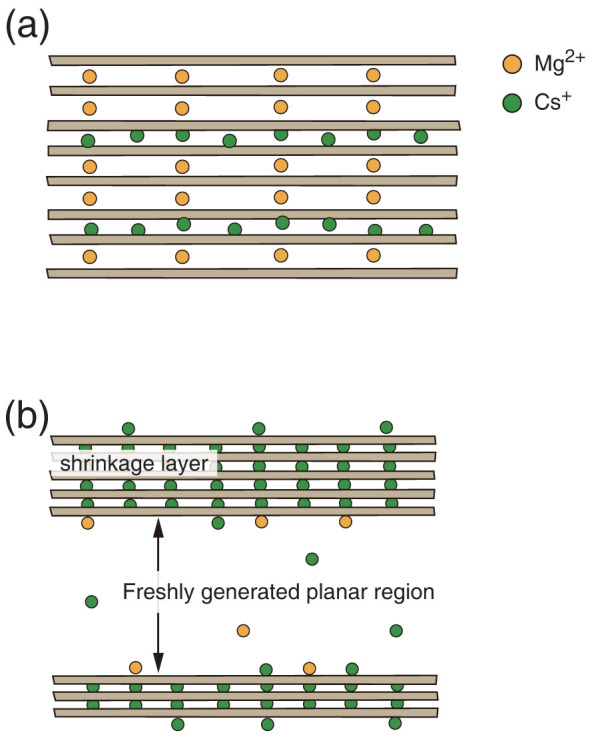
Collective structural changes induced by Cs^+^ adsorption to vermiculite clay. Schematic illustrations of the crystal domain of vermiculite clay with Cs^+^. (a) Collective intercalation (localization) of Cs^+^ in the selective layer spaces and (b) segmentation of the crystal domain of vermiculite clay, providing fresh planar adsorption sites for Cs^+^.

The dashed line in Fig. 3b is a single exponential function, exp(-*w*_Cs_/*w*_Cs_*), which roughly reproduces the change in *N* with *w*_Cs_, where *w*_Cs_* = 0.39. Thus, the segmentation gradually starts well below *w*_Cs_*/10 ≈ 0.04 mg (thick arrow, Fig. 3b). Once one partially dehydrated Cs^+^ exchanges with a hydrated Mg^2+^ in a vermiculite interlayer space, the other Cs^+^ is likely to occupy the neighboring adsorption site in the same layer space. If Mg^2+^ occupies the neighboring site to an intercalating Cs^+^, a difference in the layer spaces arises at the neighboring adsorption sites increasing the local strain energy of the clay sheet compared with Cs^+^ occupancy. Furthermore, Okumura and coworkers examined the adsorption mechanism of Cs^+^ in clay minerals by high-resolution transmission electron microscopy imaging in mica. They reported that some Cs ions are collectively located in the layer spaces and did not exchange individually with a hydrated Mg^2+^^39^. Based on these observations and our results, the adsorption behavior of Cs^+^ in vermiculite clay in suspension can be well characterized by two competitive phenomena: (i) localization of partially dehydrated Cs^+^ in the selective interlayer spaces caused by collective adsorption (Fig. 4a) and (ii) segmentation of the crystal domain (Fig. 4b) shown in the SAXS profiles, that provides fresh planar adsorption sites for Cs^+^. The contributions of (i) and (ii) should account for the intriguing structural changes in the vermiculite/Cs^+^ suspension.

Vermiculite clays are ubiquitous in the soil around Fukushima^40^, and they exhibit high affinity for adsorbing radioactive cesium compared with other minerals. Our work should help to predict the environmental fate of radioactive cesium in the polluted soils at Fukushima, which is very important for radiation protection and decontamination.

## Methods

### Materials

Vermiculite clay from Ishikawa-Gun, Fukushima, Japan, extracted from the soil, was purchased from Geo-Science Materials Nichika Co., Ltd. (Kyoto, Japan). Details of this material are available elsewhere^41^. Reagent grade CsCl was purchased from Wako Pure Chemical Industries, Co., Ltd. (Osaka, Japan) and used without further purification. The water used in this study was deionized with a Milli-Q purification system (Merck Millipore, Billerica, MA).

### X-Ray fluorescence measurements

The chemical composition of vermiculite clay was determined with an X-ray fluorescence (XRF) spectrometer (ZSX Primus II XRF, Rigaku Co. Ltd., Tokyo, Japan). Measurements took 20 min and were conducted at room temperature in vacuo. Dry, powdered vermiculite clay powder was placed on a 10 mm sample plate (Cat. No. RS540-10, Rigaku), and a small amount of powder was packed into a special cell (Cat. No. 33990051, Rigaku) for quantitative analysis of the atomic composition. The chemical composition of vermiculite used in this study was evaluated as (Mg_0.41_Ca_0.01_K_0.01_)(Mg_2.55_Ni_0.01_Fe_0.42_ Cr_0.01_Al_0.01_)(Si_2.71_Al_1.29_)O_10_(OH)_2_.

### X-ray diffraction measurements

A diffractometer (M03XHF22, MAC Science Co., Ltd., Tokyo, Japan) was used to obtain the X-ray diffraction (XRD) data for vermiculite powder to confirm its purity. The measurements were performed at room temperature with a Cu-Kα line generating an incident wavelength of 1.54 Å and with an angle (*θ*) step of 0.04° for 2*θ* = 2 to 120°. Vermiculite clay was crushed briefly in a mortar before the XRD measurements. The XRD profile is shown in the supplementary information. The peak position originates from the interference between the 2:1 phyllosilicate clay sheet structures and corresponds well to the previous results for vermiculite containing hydrated Mg^2+^ (verm-Mg) in the interlayer spaces^18^,^26^. The peak is indicated by thick arrows in supplementary data 1. The average distance between the neighboring stacked clay sheets is calculated as 14.4 Å from the peak position. Because no impurities are observed in the XRD profile, the vermiculite clay is very pure. The atomic composition of vermiculite and its XRD profile suggest that, initially, hydrated Mg^2+^ occupies the interlayer space.

### Sample preparation for SAXS

Vermiculite clay (2 g) in water (50 mL) was sonicated with an ultrasonicator (XL2020, Misonix Co., Ltd., Farmingdale, NY) in pulse mode at 80% intensity for 60 min to disperse the verm-Mg clay. Vermiculite clay was collected by filtration and then dried at 40°C. The dried powder (200 mg) was dispersed in aqueous CsCl solution (20 mL) at five Cs^+^ concentrations, ranging from 1 to 1000 ppm (Table 1) for one month. The range of pH values for sample 1 to 5 at 25°C was between 6.41 and 6.89 after one month (see Table 1). Each suspension was directly measured by SAXS.

After reaching adsorption equilibrium between Cs^+^ and vermiculite clay, the amount of Cs^+^ adsorbed to the vermiculite clay was evaluated by ICP-MS (NexION 300D, PerkinElmer Co., Ltd., Kanagawa, Japan), as follows. First, the suspension was centrifuged at 8000 rpm for 15 min and the vermiculite containing adsorbed Cs^+^ precipitated. The supernatant liquid was sampled and diluted with 0.1 N HNO_3_, filtered through a cellulose nitrate membrane (0.45 μm; Merck Millipore), and analyzed by ICP-MS to determine the Cs^+^ concentration ([Cs^+^]_sup_). The values of *w*_Cs_ and the weight rate (%) of Cs^+^ adsorption to vermiculite with respect to [Cs^+^]_ini_ (*ϕ*_Cs_; see Table 1) were calculated from [Cs^+^]_sup_. In addition to Cs^+^ detection by ICP-MS, Mg^2+^ in the supernatant liquid was confirmed in all of the samples. The concentration of Mg^2+^ in the supernatant liquid gradually increased from sample 1 to 5. This implies that Cs^+^ adsorption to vermiculite clay occurs through the ion-exchange mechanism.

### SAXS experiments

SAXS measurements were performed using X-ray diffraction apparatus (NANO-Viewer, Rigaku). The incident X-ray beam was generated from a Cu-Kα line (wavelength *λ* = 1.54 Å) and was focused to a spot 450 μm in diameter at the sample position with a confocal optic (Max-Flux, Rigaku) equipped with a pinhole slit collimator. The scattered X-rays from the sample were detected by a two-dimensional position-sensitive detector (Pilatus 100K/R, Rigaku), with 195 × 487 pixels (33.5 × 83.8 mm) and a spatial resolution of 0.172 mm, covering a *q* range of (0.02 Å < *q* < 0.5 Å^−1^) at a sample-to-detector distance of 325 mm. The scattering data recorded on the detector were corrected for counting efficiency, instrumental background, and air scattering on a pixel-to-pixel basis. The X-ray scattering intensity distribution, *I*(*q*), was circularly averaged. The sample solutions were loaded into glass capillary cells with 0.01-mm-thick walls and a 2.0 mm sample thickness. All X-ray scattering data were acquired at 25°C. Cell scattering and solvent (H_2_O) scattering were subtracted from *I*(*q*) by considering the transmission and the volume fraction of H_2_O in the sample solution.

### SAXS data analyses

To examine the effect of the Cs^+^, the SAXS profiles were evaluated by the following method. Because the vermiculite samples were unlikely to disperse homogeneously in the glass capillary cell during the SAXS measurements owing to the specific gravity, the scattering intensity was not plotted on a relative scale. To overcome this limitation, *I*_obs_(*q*) was normalized to *Q*^30^,^31^, which allowed the scattering profiles to be compared on a relative intensity scale (see equation (1)). To calculate *Q*, the upper cutoff for the integration was defined as *q*_max_ = 0.5 Å^−1^ instead of infinity, where *q*_max_ = 0.5 Å^−1^ is sufficiently large for the continuum approximation.

The finite resolution of the instruments can be incorporated by convoluting the ideal scattering intensity and the resolution function, which was approximated by a Gaussian profile of the standard deviation, Δ*q* = 0.0051 Å. The value of Δ*q* used in this study was calculated in accordance with the literature^42^. *P*(*q*) in equation (3) varies slowly with *q* in the *q*-region, whereas *S*(*q*) produces a sharp peak and varies rapidly. Therefore, the effect of finite instrumental resolution is used for only the structure factor.

The distribution of *R* had to be considered to reproduce the scattering intensities precisely, so that the Schultz distribution with the corresponding standard deviation, *σ*_R_, was used, which could contribute to enhance computation rate^43^. In equation (4), the distribution of *D* was also included based on the Gauss distribution with the standard deviation *σ*_T,*i*_, which reflected the calculations with equation (6).

## Author Contributions

R.M. and H.E. designed and led the research, carried out experiments and data analysis, and documented the findings; R.M., H.E. and S.Y. wrote the manuscript and prepared the figures. S.N. designed the small-angle X-ray scattering experiments. T.K., S.S. and T.Y. participated in scientific discussions. All authors contributed to the interpretation of results and to the finalization of the submitted manuscript.

## Supplementary Material

Supplementary Information XRD profile of dry vermiculite powder.

## Figures and Tables

**Table 1:** Compositions determined by ICP-MS for suspensions of vermiculite with Cs^+^

Sample No.	[Cs^+^]_ini_ (ppm)	*w*_verm _(mg)	*w*_Cs_ (mg)	*ϕ*_Cs_ (wt %)	pH
1	1	200	0.018	90.1	6.76
2	5	200	0.099	98.7	6.89
3	20	200	0.384	96.1	6.72
4	100	200	1.97	95.1	6.45
5	1000	200	6.94	34.7	6.41

**Table 2:** Characteristic parameters determined by model analysis of SAXS for aqueous suspensions of vermiculite with Cs^+^

Sample No.	*R* (Å)	*σ*_R _(Å)	*N*	*f*_1_	*f*_2_	*D*_1 _(Å)	*D*_2 _(Å)	*σ*_T,1 _(Å)	*σ*_T,2 _(Å)	*σ*_D,1 _(Å)	*σ*_D,2 _(Å)
1	168 ± 4.4	33.1 ± 2.9	90.4 ± 14.3	0.73	0.27	14.9 ± 0.1	33.5 ± 0.5	0.44 ± 0.07	5.97 ± 0.72	4.37 ± 0.04	11.5 ± 0.4
2	164 ± 4.5	28.7 ± 2.6	98.8 ± 20.4	1	0	14.9 ± 0.1	–	0.50 ± 0.07	–	3.25 ± 0.04	–
3	169 ± 4.3	31.5 ± 2.3	91.8 ± 16.4	1	0	14.9 ± 0.1	–	0.44 ± 0.09	–	3.37 ± 0.04	–
4	166 ± 4.5	25.5 ± 2.7	53.7 ± 7.9	0.79	0.21	14.7 ± 0.1	14.0 ± 0.2	0.55 ± 0.08	1.50 ± 0.10	4.03 ± 0.05	0.54 ± 0.1
5	165 ± 8.1	83.1 ± 4.8	25.5 ± 3.5	1	0	12.6 ± 0.3	–	1.66 ± 0.25	–	4.48 ± 0.10	–
