# Supplementary material for: Collective Structural Changes in Vermiculite Clay Suspensions Induced by Cesium Ions
Source: Sci Rep. 2014 Oct 10;4:6585. doi: 10.1038/srep06585 (PMC4192615; doi:10.1038/srep06585)
Supplement: Supplementary Information — XRD profile of dry vermiculite powder. [file srep06585-s1.pdf]

SUPPLEMENTARY FILE

Collective Structural Changes in Vermiculite Clay Suspensions Induced by Cesium Ions

Ryuhei Motokawa[1], Hitoshi Endo[2,3], Shingo Yokoyama[4], Shotaro Nishitsuji[5], Tohru Kobayashi[1], Shinichi Suzuki[1] & Tsuyoshi Yaita[1]

[1]*Actinide Coordination Chemistry Group,Quantum Beam Science Directorate (QuBS), Japan Atomic Energy Agency (JAEA), Tokai, Ibaraki 319-1195, Japan.*

[2]*Neutron Science Division, Institute of Material Structure Science, High Energy Accelerator Research Organization, 203-1 Shirakata, Tokai, Ibaraki 319-1106, Japan.*

[3]*Department of Material Structure Science, The Graduate University for Advanced Studies (SOKENDAI), 203-1 Shirakata, Tokai, Ibaraki 319-1106, Japa.*

[4]*Central Research Institute of the Electric Power Industry, Abiko, Chiba 270-1194, Japan.*

[5]*Graduate School of Science and Engineering, Yamagata University, Yonezawa, Yamagata 992-8510, Japan.*

*To whom correspondence should be addressed.

Dr. Ryuhei Motokawa,

E-mail: motokawa.ryuhei@jaea.go.jp

Tel & Fax: +81-29-284-3747

Dr. Hitoshi Endo,

E-mail: hitendo@post.j-parc.jp

Tel & Fax: +81-29-284-4720

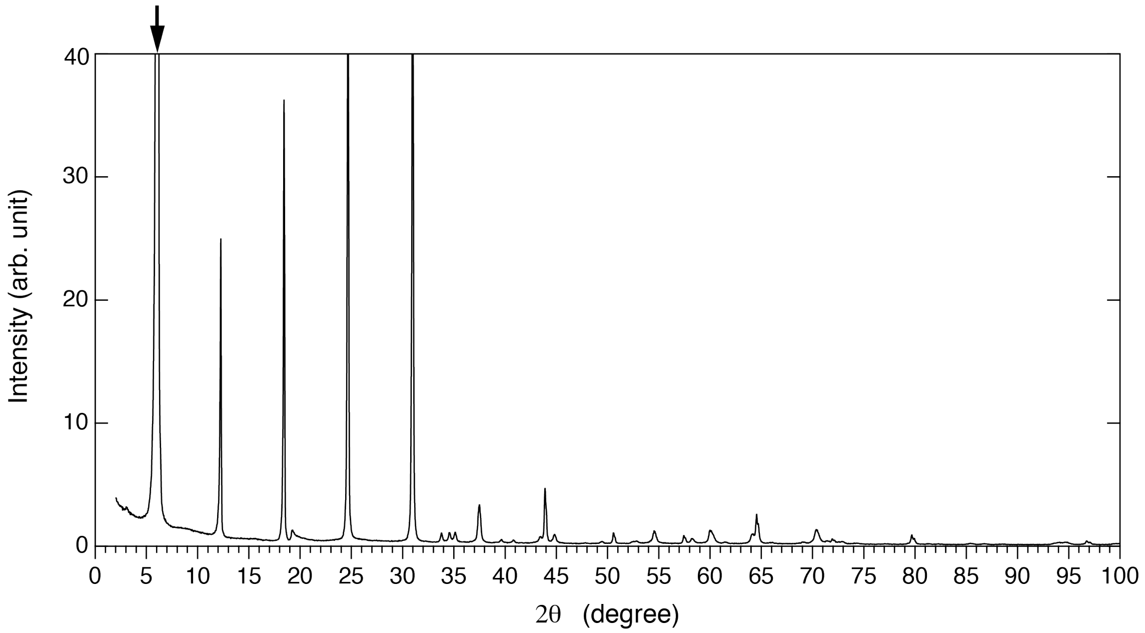

Supplementary Data 1. | XRD profile of dry vermiculite powder. XRD profile of vermiculite powder used in this study at room temperature in air.
